# Adapting the Use of Digital Content to Improve the Learning of Numeracy Among Children With Autism Spectrum Disorder in Rwanda: Thematic Content Analysis Study

**DOI:** 10.2196/28276

**Published:** 2022-04-19

**Authors:** Theoneste Ntalindwa, Mathias Nduwingoma, Alphonse Uworwabayeho, Pascasie Nyirahabimana, Evariste Karangwa, Tanjir Rashid Soron, Thomas Westin, Thashmee Karunaratne, Henrik Hansson

**Affiliations:** 1 School of Education University of Rwanda Kigali Rwanda; 2 School of Inclusive and Special Needs Education University of Rwanda Kigali Rwanda; 3 Telepsychiatry Research and Innovation Network Ltd Dhaka Bangladesh; 4 Department of Computer and Systems Sciences Stockholm University Stockholm Sweden

**Keywords:** autism, learning, ICT, e-learning, education, children, ASD, teaching, teachers, communication, communication technology, online content, Rwanda, gamification, school, school-age children, behavior

## Abstract

**Background:**

Many teachers consider it challenging to teach children with autism spectrum disorder (ASD) in an inclusive classroom due to their unique needs and challenges. The integration of information communication technology (ICT) in the education system allows children with ASD to improve their learning. However, these ICT tools should meet their needs to lead a productive life.

**Objective:**

This study aimed to examine the possibilities of re-creating and adapting digital content to improve the learning of numeracy among children with ASD in inclusive school settings.

**Methods:**

We conducted 7 focus group discussions (FGDs) with 56 teachers from 7 schools and 14 parents from April to November 2019. Each of the FGDs took around 1 hour. Two clustered sets of questions were used: (1) general knowledge about teaching children with ASD and (2) analysis of selected online educational video content of early math (specifically, counting numbers). The researchers used video to understand current methodologies used in teaching children with ASD, possibilities of adaptation of the content in the current teaching environment, future challenges when the content is adapted, and possible solutions to overcome those challenges. All data, including audio recordings, field notes, and participants’ comments, were transcribed, recorded, and analyzed following the steps recommended in qualitative data analysis.

**Results:**

The researchers identified ten themes from the analysis of the data: (1) awareness of the existence of ASD among children in schools and the community, (2) acceptance of children with ASD in an inclusive classroom and the community, (3) methods and models used when teaching children with ASD, (4)realia used to improve the learning of children with ASD, (5) the design of educational digital content, (6) the accessibility of online educational content, (7) quality of the content of the educational multimedia, (8) the opportunity of using the translated and re-created content inside and outside the classroom, (9) the relevance of the digital content in the Rwandan educational system, and (10) enhancement of the accessibility and quality of the digital content. We found that participants assumed that the content translation, gamification, and re-creation would help teach children with ASD. Moreover, they recommended contextualizing the content, increasing access to digital devices, and further research in the education of different subjects.

**Conclusions:**

Although many studies have identified the possibilities of using ICT to support children with ASD, few studies have documented the possibilities of integrating the existing technologies tested in the international community. This study is charting new territory to investigate online content to suit the context of schools. This study recommends further exploration of possible methodologies, such as applied behavior analysis or verbal behavior therapy, and the development of contextualized technologies that respond to the educational needs of children with ASD.

## Introduction

Autism spectrum disorder (ASD) is a neurodevelopmental disorder that appears during the developmental period when the accommodation process starts [1]. The prevalence of autism has increased in recent years, and many people with this condition live in low- and middle-income countries, while only about 1% of research studies have represented these countries in the past decade [2]. The situation is especially disappointing for children with autism, who have specific needs and different behavioral patterns in sub-Saharan Africa than in developed countries.

Children with disabilities face challenges when learning in an inclusive environment [3]. These challenges become critical for children with ASD [4]. More than half of children with learning disabilities enrolled in primary education in Rwanda drop out of school at an early age [5].

According to the National Commission for Persons with Disabilities (NCPD) and National Commission for Children (NCC) [6], few centers care for children with disabilities, because the cost of education for children with ASD is still high, making it difficult for them to continue with their studies. Some schools and centers are still facing the problem of accessing information communication technology (ICT) tools to improve the learning of children with ASD. However, a good number of primary education schools are equipped with computers obtained through initiatives such as One Laptop per Child [7] and the Computer on Loan scheme [8]. These initiatives created the opportunity to use technology to enhance students’ learning regardless of their level of learning ability. Several studies [9-12] have shown that ICT integration in the classroom supports children with ASD and other developmental disabilities.

The Rwanda Ministry of Education [13] has developed a competence-based curriculum framework with 7 key competencies: literacy, numeracy, ICT, citizenship and national identity, entrepreneurship and business development, science and technology, and communication in the official languages. Literacy and numeracy are basics to accessing learning in other subjects. The Rwanda competence-based curriculum states that all children must be equipped with skills in computing at the end of the basic education program. They should also be able to use numerical patterns and relations to solve problems related to everyday activities, for example, in commercial contexts and financial management, and interpret basic statistical data using tables, diagrams, charts, and graphs [14].

Children with ASD may enjoy basic arithmetic if the content is gamified using multimedia [10]. According to Spek et al [11], gamification of content for the learning of children with ASD brings the opportunity to overcome limitations on accessibility by fostering independence and assisting the children in social relationships. Moreover, ICT has made the internet one of the best resources for discovering entertaining activities that teach and excite children with different learning disabilities.

Educational websites such as IXL Worldwide [12] and Khan Academy [15] provide content to support the learning of children with learning disabilities, including dyslexia, dysgraphia, attention deficit and hyperactivity disorder, and visual-motor deficit. The introduction of gamification in Khan Academy videos has proved promising to bring new ways of engaging students with activities and providing valuable data for teachers [15,16]. Moreover, several studies [17-19] have shown that online content for teaching mathematics positively impacted a variety of learning environments. However, there is no research on using digital tools for intervention in children with ASD to improve their learning of numeracy skills.

Studies by Abubakar [20] and Onaolapo [21] indicate that few studies have focused on technology and education in ASD in sub-Saharan Africa. Thus, existing online content has yet to be investigated for its ability to support the learning of children with ASD in Rwanda. This study aimed to analyze how to adapt the available digital content to enhance numeracy learning for children with ASD in inclusive school settings.

## Methods

### Design and Setting

This thematic content analysis study was conducted from April to November 2019 to examine how to adapt digital content to improve learning for children with ASD in Rwanda by developing a tailor-made educational mobile app [22]. The researchers recruited 70 participants, including 56 teachers and 14 parents, from a total of 7 schools, including 5 in urban areas and 2 in rural areas. Only 2 of the urban schools were in Kigali, the capital of Rwanda: Autisme Rwanda and Groupe Scolaire (GS) Jabana; the remaining 3 were from different urban areas in upcountry provinces: HVP Rwamagana (Eastern Province) and Ubumwe Community Center (Western Province). College des Amies de la Paix du Christ Roix (APAX), Janja (Northern Province), and APAX Muramba were in the country’s rural areas.

Within the group of teachers, there were 32 males and 24 females, and in the group of parents, there were 6 males and 8 females. The teachers taught subjects including mathematics, elementary science and technologies, social sciences, and languages.

The researchers selected the schools based on data from the National Commission of Persons with Disabilities [6] and our previous study [9]. The parents were from the Rwanda Parent’s Initiative on Autism [23]. The parents were not required to have a child enrolled in any school, but might have at least one child with ASD. The inclusion criterion for the schools was that the school had a program to care for children with any disability.

### Data Collection

The researchers used focus group discussions (FGDs) to collect the data for this study. FGDs were conducted in a separate room in each school to provide an environment where parents could give accurate, complete, and sincere answers during the discussion. The questions in the interview guide fell into two categories: (1) general knowledge about teaching children with ASD and (2) online video content for early math (specifically, counting numbers) (Multimedia Appendix 1). The researchers selected online content from Khan Academy, which focused on early counting mathematics, based on its audiovisual features, as these can aid learning for children with ASD. The researchers projected the selected educational video on a screen, and the participants discussed the design layout and possible modifications to adapt it to the education of children with ASD in the Rwandan context. The parents participated only in discussing the online video content for early math. The researchers chose to mix the questions according to the mission of the visited school or center. The interviews lasted for 1 hour; records and related soft copies were kept confidential and were saved on a physical drive. In contrast, hard copies of the signed consent forms were kept safe in the principal investigator’s office.

### Data Analysis

In the analysis of the data, the researchers followed the six steps of thematic content analysis recommended by Jugder [24] and Caulfield [25]: (1) familiarization, (2) coding, (3) generating themes, (4) reviewing themes, (5) defining and naming themes, and (6) writing up. The researchers used conceptual reliability to ensure the validity and reliability of the data at every step of the analysis of the coded data by involving 18 teachers and 6 parents in coding and reviewing data [26].

We followed inductive, descriptive thematic analysis of the FGD transcripts, following several steps. Firstly, transcripts were read and reread to establish familiarity with the data. Then, initial codes that captured features of interest and importance to the research questions were identified. Next, a coding framework was developed by collating initial codes to create candidate codes that meaningfully described the overall patterns of participant responses in the data. Next, researchers and representatives of the participants reviewed the transcripts to ensure that the coding framework captured participant responses. The team then coded the transcripts line by line to collate all instances of patterns identified in the data. Finally, the team collapsed the candidate codes to produce higher-order themes that were then read for patterns of similarity and divergence within and across each theme.

In the coding and review of themes, the researchers, teachers, and parents answered the following questions: (1) Did we skip any relevant information? (2) Do these themes represent the data? and (3) Could any modifications make the theme better? In this process, we split some themes and created new ones to make them more valuable and accurate. Only researchers wrote up the final manuscript, which was the last step of data analysis. Data management was supported using the qualitative software program NVivo (version 9; QSR International) [27]. Participant demographic data were analyzed using SPSS-23 (IBM) [28].

### Ethics Approval

Trained researchers from the University of Rwanda collected the data from the participants. The research project passed through the college ethical committee by following the established process [29]: (1) presentation of the research proposal, (2) submission of the application and tools to be used for ethical research clearance, and (3) review and approval of the application by the ethical research committee (review number 01/P-CE/635/EN/gi/2019) (Multimedia Appendix 2). The researchers also obtained formal informed consent from the parents and teachers using the approval form (Multimedia Appendix 3).

## Results

### Participant Characteristics and Themes Identified by the Analysis

Table 1 and Table 2 show the demographic characteristics of the parents and teachers, respectively, who participated in the study.

From the thematic analysis of the interview transcripts captured during 7 FGDs, the researchers identified the following themes: (1) awareness of the existence of ASD among children in schools and community, (2) acceptance of children with ASD in an inclusive classroom and in the community, (3) methods and models used when teaching children with ASD, (4) realia used to improve the learning of children with ASD, (5) the design of educational digital content, (6) accessibility of online educational content, (7) quality of the content of the educational multimedia, (8) opportunity of using the translated and re-created content inside and outside the classroom, (9) the relevance of the digital content in the Rwandan education system, and (10) enhancement of the accessibility and quality of the content.

**Table 1:** Sociodemographic characteristics of the parents by location.

Characteristics		Kigali City	Eastern Province	Southern Province	Northern Province	Western Province
**Gender, n**						
	Male	2	1	1	1	1
	Female	4	1	1	1	1
	Total	6	2	2	2	2
**Age, n**						
	Less than 20 years	0	0	0	0	0
	Between 20 and 25 years	0	0	0	0	0
	Between 25 and 30 years	0	0	0	0	0
	More than 30 years	6	2	2	2	2
	Total	6	2	2	2	2

**Table 2:** Sociodemographic characteristics of the teachers by school.

Characteristics		HVP–Gatagara Kigali	HVP–Rwamagana	APAX–Janja	APAX–Muramba	Ubumwe Community Center	Autisme Rwanda		GS Jabana	
**Gender, n**										
	Male	2	2	4	3	2	5		6	
	Female	6	6	4	5	6	3		2	
**Age, n**										
	Less than 20 years	1	0	1	2	1	0		0	
	Between 20 and 25 years	5	6	5	4	2	3		3	
	Between 25 and 30 years	2	1	1	1	1	3		2	
	More than 30 years	0	1	1	1	4	2		3	
	Total	8	8	8	8	8	8		8	
**Years of experience, n**										
	Less than 5 years	4	4	5	7	8	8		5	
	Between 5 and 10 years	2	3	3	1	0	0		3	
	Between 10 and 15 years	1	1	0	0	0	0		0	
	More than 15 years	1	0	0	0	0	0		0	
	Total	8	8	8	8	8	8		8	
**Subjects taught, n**										
	Mathematics	5	3	4	6	3	4		3	
	Science related	1	2	1	1	2	2		2	
	Social sciences	0	2	1	0	0	1		1	
	Languages	2	1	2	1	3	1		2	

### Awareness of the Existence of ASD Among Children in Schools and the Community

When asked about the presence of ASD among children registered in their classes, 24 of the 56 teachers (43%) reported that they were not aware of ASD among their students because they manifest different behaviors. ASD is a spectrum, and can range from low to high functioning ASD; not all children with ASD are the same. Unfortunately, this makes educators confuse children with ASD with children with other cognitive disabilities.

ASD is not yet well known in the Rwandan community, which results in various misunderstandings of the behavior of children with ASD in families. Eight teachers suggested that diagnosing children before they begin school could facilitate them in identifying teaching methods and content for children who may have ASD. One parent reported never knowing what ASD was before confirming his child’s diagnosis at a hospital. The increase of qualified medical personnel in all hospitals with professional diagnostic tools and collaboration with educators is a solution to raise awareness of ASD in schools to prepare personalized learning materials for children on the autism spectrum.

### Acceptance of Children With ASD in an Inclusive Classroom and Community

In the sample of teachers who participated in the FGDs, 32 of 56 (57%) teachers reported difficulties teaching children with ASD in the same classroom as children without cognitive disabilities. The behavior of children with ASD is a factor that creates difficulties in accepting children with ASD in all schools. However, some participants reported including children with ASD in schools when educators received training in special education for children with ASD and personalized teaching material. The teachers also said that trained educators and teaching aids were more effective if they integrated assistive technologies into their teaching and learning activities. The integration of assistive technologies such as with ICT when teaching children with ASD could bring greater success to the inclusion of children with ASD in the Rwandan education system.

### Methods Used When Teaching Children With ASD

This theme elucidated models used to support children with ASD in the educational environment. Teachers understood how to apply the applied behavior analysis model in a classroom environment and were trying to implement strategies to motivate children with ASD to stay focused while learning. Eight of 56 teachers (14%) reported that children with ASD needed quick motivations, such as giving them specific objects that they liked that were available in the school environment. Teachers could also provide personalized content to aid learning for children with ASD. For example, when asked what methods helped pupils get to grips with mathematics, teachers reported that games were the best approach to stay focused and engaged in the classroom. The introduction of games brought freedom to the children to work on the given task and join different groups of children. This approach enabled them to socialize with others and enhanced teamwork in inclusive class settings. Most teachers reported that providing personalized content and creating an environment conducive to learner interaction could help reveal the learning abilities of children with ASD and develop their inner talents.

### The Use of Realia to Improve the Learning of Children With ASD

This theme involved using various objects to provide contextualization in the education of children with ASD. Providing a connection to the school’s physical environment allowed students to better understand the content of the subject they were learning. When asked about the objects used in teaching children with ASD, 40 of 56 teachers (71%) responded that they used real examples of objects available in schools to replace the models in the curriculum syllabus. The strategy of contextualization of content helps children to understand the topic planned in the curriculum.

All teachers (100%) reported that educational videos were an alternative to support unconscious learning among children with ASD when they were out of class. Combining traditional teaching methods with ICT-enabled teaching methods in inclusive learning among children with and without ASD is the best approach to provide an equitable education to all children.

### The Design of Educational Digital Content

**Figure 1:**
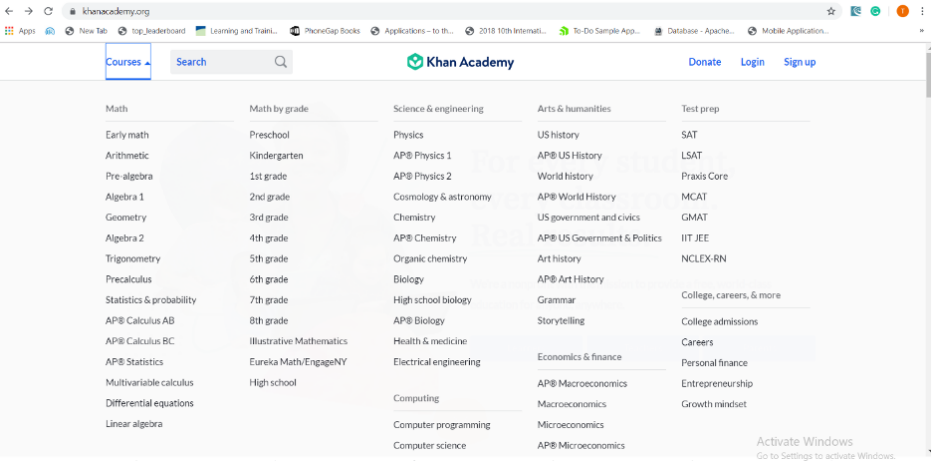
Khan Academy platform [15].

This theme explored the design of the content of the selected website. Teachers reported that it was their first time browsing the Khan Academy website, even those familiar with other websites for finding educational resources. Despite the excellent layout of the website, participants suggested explanations of some abbreviations, such as “SAT,” “LSAT,” and “MCAT,” among others. Figure 1 shows further examples of these abbreviations. All teachers (100%) appreciated the content categorization on the course page. Categorizing the cognitive level of children supports education in Rwanda from preschool to higher education. This might bring an early, positive impact when teaching children with ASD.

All 14 parents (100%) reported that their children could not follow the formal education system and recommended using the examples and images available in their families when designing a web interface for children with ASD. The contextualization of content by creating an intellectual need for information and skills could help children with ASD learn through practice to prevent them from losing attention. In addition, developing the visual and hearing senses of children with ASD could help the designers of digital content to better enable them to learn.

### Accessibility of Educational Digital Content

Access to digital content is helpful to support the education of learners with ASD. Educational videos hosted on YouTube channels are made accessible by internet connectivity. Users can download video content from YouTube and use it offline. This option is better for schools that have low internet connectivity. Among the teachers, 16 of 56 (29%) from schools in rural areas reported barriers to accessing online content due to poor internet connectivity and recommended increasing the bandwidth of their internet connection, despite the possibility of downloading the videos and playing them offline. Teachers reported that the reliability of the web interface was a driving factor for adaptation of online content for the education system in Rwanda. The 56 teachers and 14 parents also recommended developing digital content that could be accessed offline as a mobile application.

### Content Quality of Educational Multimedia

YouTube is a video-sharing platform used by many online education websites to provide educational content to various users. Professionals from other domains create the videos; some of them are published under a Creative Commons license to allow users to adapt or re-create them. In this study, participants suggested content re-creation to meet the Rwandan context. All 56 teachers (100%) reported that educational digital content could support children with ASD when designers adapt the content to the local context.

Teachers and parents argued that the translation of the content into the local language could bring a positive impact not only to the learning of children with ASD, particularly literacy and numeracy, but also to nondisabled children who are interested in using educational digital content and children who are not registered in schools. This strategy is crucial, because 13 of 14 parents (93%) did not know English, and 42 of 56 teachers (75%) had difficulty using English. The translation and re-creation of content to be adapted for the local context might be an excellent approach to integrate ICT into the education system and improve the learning of children with various cognitive disabilities in Rwanda.

### Opportunities to Use Translated and Re-created Content Inside and Outside the Classroom

This theme elucidated the opportunity to use the re-created and translated content to upgrade the current education system to international standards. All 56 teachers and 14 parents reported that Khan Academy’s content could help teach basic mathematics to children with ASD because of the availability of interactive videos. In the parent group, 13 of 14 (93%) reported that the content was useful in helping their children be engaged when they were at home. Teaching children with ASD in the local language has greater advantages than teaching in a foreign language, as this creates a deficit in communication. In addition, the translation and adaptation of international content can bring new opportunities to all children to learn the same content prepared by globally recognized experts in different fields.

### The Relevance of the Digital Content in the Rwandan Education System

Of the 70 total participants in this study, all the teachers (56/70, 80%) and parents (14/70, 20%) welcomed the implementation of technology-enhanced content in teaching. The teachers reported that digital educational material was essential in preventing cognitive overload and providing content. However, ICT adoption in education needs strategies and financial investment to obtain positive results. Among the teachers, 16 of 56 (29%) suggested that these strategies could include training educators in digital content in teaching and learning, as well as improving infrastructure. The availability of offline content is considered a solution to bridge the gap in internet access between urban and rural areas in Rwanda. However, this is not a perfect solution, as some schools still report a lack of digital devices to play the content offline. Adopting mixed digital and traditional learning methods could enable children to learn despite a lack of digital devices.

### Enhancement of the Accessibility and Quality of the Content

**Figure 2:**
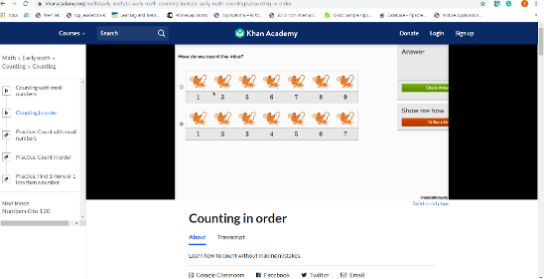
Interface for early math (counting) [17].

In the FGDs, 65 of 70 (93%) participants suggested further online teaching for children with ASD. Teachers criticized the researchers’ content during a contextualization exercise for teaching early mathematics [30] in data collection. The narrator’s voice in the videos was also not well understood by the teachers. The participants suggested replacing the narrator with a speaker of a local language. The teachers and parents suggested the re-creation of the content to add examples of situations that the children encounter in their everyday lives. Figure 2 shows the interface for early math (counting).

Teachers suggested that re-creating the content of this lesson to better suit the Rwandan teaching environment would provide personalized material to improve the learning of children with ASD in an inclusive environment. Participants also recommended a subscription model for the re-created content to improve its accessibility.

## Discussion

### Principal Results

The findings of this study indicate the possibility of using digital content in the education of children with ASD. The themes reported in the results section show that integrating multimedia content can increase access to education for children with ASD [9]. This is possible if educators are aware of children with ASD in their schools and adopt methodologies that help these learners stay focused [31]. This could increase the acceptance of these children in mainstream schools [18].

The development of various models to support children with ASD [32], as well as computer-assisted educational content [19], is progressing rapidly in various domains. Nevertheless, such research needs to computerize existing methodologies and content in an individualized context. A study by O’Malley et al [33] reported that educational software for children with ASD must respond to specific interests and developmental needs. In addition, the interface for such software must be as interactive as possible to most effectively facilitate learning in children with ASD [34].

Teaching basic numeracy to children who have a deficit in communication, such as those with ASD, is more possible when the content is gamified using multimedia technologies [16]. All participants in this study used games when teaching various subjects in class. Educational videos are essential to improve the learning of children with ASD [35,36] (Multimedia Appendix 4). After watching the Khan Academy content used in this study [17], the teachers reported that integrating content after it was re-created could allow learners with ASD to participate in an inclusive classroom [30].

This study evaluated Khan Academy content [17] on the YouTube platform [37], which has gamified content delivered in educational institutions. A study by Baker et al [38] found that integrating multimedia content improved learning for various subjects [39]. Participants in the present study suggested re-creating the Khan Academy content to meet the school environmental context.

This study was carried out before the COVID-19 pandemic became a crisis in Rwanda [40]. However, our results demonstrate that online content could help educate children with ASD during and after the COVID-19 crisis. A study by Stenhoff et al [41] documented the possibility of supporting children with ASD through distance education during school closures. The availability of digital content for learners with ASD is also crucial to support remote learning in response to COVID-19 prevention measures [42].

The translation of the findings of this study into practice would be helpful for educators and would enable future research to address barriers to education for children with ASD by focusing on the functional abilities of these children, rather than using a deficit model based on specific diagnoses. Furthermore, enabling children with ASD to learn mathematics would contribute to eliminating all causes and obstacles that can lead to disparity in education, such as gender, disability, and geographical or social group. This is an objective of the Rwanda education sector [14].

### Strengths and Limitations

This study had many strengths. It reflects the experiences of teachers who serve to support children with ASD; it was performed by experienced researchers in education, information technology, special education, and ASD; and it included schools from both rural and urban areas. Thus, our findings might allow teachers to adopt new, different methods and innovative tools to improve teaching and learning. Nevertheless, this study was limited by including only a small number of teachers and parents, making our results hard to generalize to all education practitioners. Furthermore, we only focused on the subject of basic numeracy, whereas primary education includes many more subjects.

In future studies, all content relating to the existing syllabus should be explored, and participants should be allowed more time to test the interface of the software and provide more detailed opinions. In addition, a longitudinal usability study of the interface might help uncover long-term advantages and disadvantages that teachers may experience and enable adaptation of the curriculum in a way that the first-time experiences described here could not. Finally, future research should further explore the development of personalized ICT solutions for individuals with ASD that respond to their educational needs.

### Conclusions

The study documented the process of contextualization of technology to make it a better solution that meets the actual context of its environment. Integrating systems designed by internationally recognized experts and translating these systems into a local context could bring innovation in teaching children with disabilities. This study charts new territory in the investigation of online content and its ability to match the context of primary and secondary schools. We recommend further exploration of methodologies such as applied behavior analysis and verbal behavior therapy, and we also recommend the development of contextualized technologies that respond to the educational needs of children with ASD.

## Appendix

Multimedia Appendix 1 Interview guide question for primary teachers.

Multimedia Appendix 2 Research Ethical Clearance.

Multimedia Appendix 3 Informed Consent Form for teachers.

Multimedia Appendix 4 Sample Form for Observers’ Notes at Focus Groups.

## Abbreviations

ASD: autism spectrum disorder
FGD: focus group discussion
ICT: information and communication technology
