# Supplementary material for: Adapting the Use of Digital Content to Improve the Learning of Numeracy Among Children With Autism Spectrum Disorder in Rwanda: Thematic Content Analysis Study
Source: JMIR Serious Games. 2022 Apr 19;10(2):e28276. doi: 10.2196/28276 (PMC9066332; doi:10.2196/28276)
Supplement: Multimedia Appendix 1 [file games_v10i2e28276_app1.pdf]

Multimedia Appendix 1: Interview guide question for primary teachers

We are working on a study to support teaching math to children with Autism in schools.

1. Do you have some children with Autism in your school?
2. Describe your teaching method – how would it help pupils get to grips with math?
3. How do you plan to use technology in a typical math lesson?
4. How would you boost a pupil's attention when you are teaching math?

We are evaluating the interface of Khan Academy to support teaching numeracy children with Autism.

Let us watch the short course from Khan Academy teaching basic counting.

https://www.khanacademy.org/math/early-math/cc-early-math-counting-topic/cc-early-math-counting/v/counting-in-order

5. How do you find this interface to help to learn of children with cognitive disabilities like those who have Autism?
6. As I introduced, the goal of this research is to translate the content of Khan Academy in the Kinyarwanda language to increase the accessibility of digital content to a person with disabilities.
   What are your suggestions to be done on the interface to meet the Rwandan context?
7. What are the challenges you find to be addressed before using this interface?
8. If the content is translated into Kinyarwanda, what are elements can be included in the interface to help children with Autism to stay focused.
9. Teaching children with Autism in class, we need reward actions for them. How do you reward the children in the class to encourage them in their learning?
10. Do you have any other comments or suggestions that may contribute to this study to close this discussion?
