# Supplementary material for: Adapting the Use of Digital Content to Improve the Learning of Numeracy Among Children With Autism Spectrum Disorder in Rwanda: Thematic Content Analysis Study
Source: JMIR Serious Games. 2022 Apr 19;10(2):e28276. doi: 10.2196/28276 (PMC9066332; doi:10.2196/28276)
Supplement: Multimedia Appendix 2 [file games_v10i2e28276_app2.pdf]

Multimedia Appendix 3: Research Ethical Clearance

UNIVERSITY of RWANDA

COLLEGE OF EDUCATION

RESEARCH AND INNOVATION UNIT

Rukara, 27th May, 2019
Ref: 01/P-CE/635./EN/gi/2019

TO WHOM IT MAY CONCERN

I am pleased to you Mr. Theoneste Ntalindwa, a citizen of Rwanda, who is currently an employee of the University of Rwanda-College of Education as E-learning Officer and a doctoral student of the School of Education at the University of Rwanda-College of Education. As a PhD by research student, he is conducting a research entitled: "**Developing educational mobile application for persons with Autism Spectrum Disorders in Rwanda**". His research will involve both children with Autism Spectrum Disorders (ASD) and their parents recruited respectively from schools and centers of children with special needs education and association of parents of children with ASD.

Mr. Ntalindwa's research project passed through an internal collegial ethical process. Thus, the University of Rwanda-College of Education: Directorate of research and Innovation confirms that this research adheres to ethical standards and principles. Therefore, we kindly request you to accord him your cooperation to enable his research to be successful.

Your permission for him to conduct the study will be highly appreciated.

Yours sincerely,

UNIVERSITY OF RWANDA - COLLEGE OF EDUCATION - CE

Assoc. Prof. Eugene Ndabaga
Director of Research and Innovation
University of Rwanda- College of Education
E-mail: ndabagav@yahoo.ie
Mobile: +250788308862

Cc:

- The Principal, UR-CE
- Dean, School of Education
- Dr Mathias Nduwingoma
