# Supplementary material for: Adapting the Use of Digital Content to Improve the Learning of Numeracy Among Children With Autism Spectrum Disorder in Rwanda: Thematic Content Analysis Study
Source: JMIR Serious Games. 2022 Apr 19;10(2):e28276. doi: 10.2196/28276 (PMC9066332; doi:10.2196/28276)
Supplement: Multimedia Appendix 3 [file games_v10i2e28276_app3.pdf]

Multimedia Appendix 2: Informed Consent Form for teachers

This informed consent form is for teachers of children participating in the research study titled: ***"Digital content as a supporting tool in the learning of numeracy among children with Autism Spectrum Disorders in Rwanda: A Thematic content analysis study"***

The study is conducted by Théoneste Ntalindwa (Ph.D. candidate at the University of Rwanda, Rwanda), Nduwingoma Mathias, Karangwa Evariste, Alphonse Uworwabayeho (University of Rwanda, Rwanda) and Tanjir Rashid Soron (Neuro-Developmental Disabilities Protection Trust, Bangladesh).

This Informed Consent Form has two parts:

- Information Sheet (to share information about the study with you)
- Certificate of Consent (for signatures if you agree that your child may participate)

Part 1: Information Sheet

Introduction

We are researching to enable people with Autism Spectrum Disorders to be included in the basic education system in Rwanda. In short, the study is about finding out how the content, interface, and process of Khan Academy can be useful to support teaching basic mathematics in Rwandan primary schools.

In our research, we need to observe teachers teaching basic mathematics, exploring their lesson plans and teachers' guides. The focus group discussion will also take place.

To do this, we ask you as a teacher for permission. After reading about the study below, and if you agree, then the next thing we will do is ask you for their agreement before the session.

There may be some words that you do not understand. In that case, please feel free to either contact us via e-mail or the principal at the school, who can send us all your questions via e-mail so we can answer them before you sign the certificate of consent.

Voluntary participation

You do not have to agree that you can participate in the study. You can choose to say no, and any services you receive at the school will not change. We know that the decision can be difficult. You can ask as many questions as you like, and we take the time to answer them via the principal. You do not have to answer any questions or participate in the observation if you feel uncomfortable doing so.

Procedure

The study will be done at the << school>>**.** You will participate in one session of about 15 minutes in focus group discussion. The interviews will be recorded.

The questions that are likely to be asked during the focus group discussion will be related to the main research question: How useful is Khan Academy content, process, and platform for supporting children with ASD learning in Rwanda?

Benefits

There will be no immediate and direct benefit to you or your school. However, your participation is likely to help us find out more about how the content of Khan Academy can be made more accessible and support the inclusion of people with neuro-developmental disabilities like ASD in the education system with other normal students. We hope that the results will help the integration of ICT in your teaching profession and enable your child to perform like others at the school, as well as improve communication between you and your pupils.

Reimbursements

You will not be provided with any payment to take part in the research. However, the interface for teaching basic mathematics will be made available for free.

Confidentiality

We, as researchers, will not share information about you. However, because something out of the ordinary is being done through research in your community, it will draw attention. If you participate, you may be asked questions by other people in the community. We cannot guarantee confidentiality, but we believe that the nature of the research question is not very sensitive or personal.

Sharing of Research Findings

We will share what we have learned with the participants and the principal. Nothing that your child will tell us during the sessions will be attributed to him/her by name. A written report will also be given to the participants, which they can share with their community. We will also publish the results in order that other interested people may learn from our research.

Right to refuse or withdraw

You may choose not to participate in this study. Choosing to participate or not will not affect your future at the school in any way. You still have all the benefits that would otherwise be available at this school. You stop participating in the observation at any time that you wish without either of you losing any of your rights.

Whom to Contact

If you have any questions, you may ask them now or later, even after the study. If you wish to ask questions, you may contact either the principal or Théoneste Ntalindwa, ntatheos@yahoo.co.uk, Telephone: 0788884594. Mathias Nduwingoma ndumathias2001@yahoo.com, Telephone: 0788897814. Evariste Karwanga karangwa81@ymail.com, Telephone: 0785489767.

Part 2: Certificate of Consent

I was a teacher and have been asked to consent to participate in this research study, which will involvee observation and a brief interview. I have read the foregoing information, or it has been read to me. I have had the opportunity to ask questions about it, and any questions that I have asked to have been answered to my satisfaction. I consent voluntarily to participate in this study.

Print Name of Teacher ___________________________________

Signature of Teacher____________________________________

Date __________________________

Day/Month/Year
