# Supplementary material for: Adapting the Use of Digital Content to Improve the Learning of Numeracy Among Children With Autism Spectrum Disorder in Rwanda: Thematic Content Analysis Study
Source: JMIR Serious Games. 2022 Apr 19;10(2):e28276. doi: 10.2196/28276 (PMC9066332; doi:10.2196/28276)
Supplement: Multimedia Appendix 4 [file games_v10i2e28276_app4.pdf]

Multimedia Appendix 4: Sample Form for Observers' Notes at Focus Groups

**FOCUS GROUP DATA CAPTURE PRO-FORMA**				
Study Title:	**Digital content as a supporting tool in the learning of numeracy among children with autism spectrum disorders in Rwanda: A Thematic content analysis stud**			
Date:				
Moderator Name:				
Name of school/center				
No. Participants:				
Start Time:				
End Time:				
**Participant Demographics:** ***enter column headings as appropriate***				
Gender	Number	Job		
Male				
Females				
Discussion Question 1				
Do you have some children with Autism in your class?				Time
1. I did not know that my kid had Autism before he got a diagnosis from a medical Doctor.<br>2. In our class is not easy to confirm whether on child has Autism because we confuse it with other learning disabilities,<br>3. *It is not easy to include children with ASD in the same classroom because those children have very challenging behavior. For example, if you are teaching and when you ask them to respond to the questions, those children refuse to respond*"				5 min
Discussion Question 2				
Describe your teaching method – how would it help pupils get to grips with math?				Time
1. *When a child does come activity, s/he can tell you any other activity he/she wish to perform. For example, here, a child can accomplish to fabric the cap, and then he/ she insists on being allowed to make a calf, for example. We allow him/her what s/he wishes to do even though s/he is not able to make it. We have seen that, if we deny it, the learner's happiness and courage disappear!*				5 min

2. *One of the methods we use is the introduction of games on each topic we are teaching. This makes children stay focused and be engaged in the class because they always enjoy games ".*<br><br>3. *Students are given a task to cut pieces of clothes. All students can't make it well at the same time. There is a time when one among the best performers asks the teacher to go and help those who fail to do the task. That time we allow that to go on at the same level. That kind of teamwork also makes the children happy.*	
Discussion Question 3	
How do you plan to use technology in a typical math lesson?	Time
1. *In the evening, we use the projector screen to play some educational videos and cartoons that bring children together, and we see improvement after a given time.*<br>2. *Like children with other disabilities like vision or hearing impairment have different tools that enable them to learn in class, I also wish the availability of tools that may bring children with ASD to stay focused when they are in the class.*<br>3. We find that digital games can help children with Autism to learn. However, most of the tools we use are in a foreign language which are hard to learn for those children.	5 min
Discussion Question 4	
How would you boost a pupil's attention when you are teaching math?	Time
1. *In such a case, we use the real examples of objects which are found in our region to replace the examples provided in the book. This strategy helps children to better understand the topic we are teaching.*<br>2.	5 min
Discussion Question 5	
We are evaluating the interface of Khan Academy to support teaching numeracy children with Autism.<br><br>Let us watch the short course from Khan Academy teaching basic counting.<br><br>**https://www.khanacademy.org/math/early-math/cc-early-math-counting-topic/cc-early-math-counting/v/counting-in-order**<br><br>The video is played three times	5 min
Discussion Question 6	
How do you find this interface to help to learn of children with cognitive disabilities like those who have Autism?	5 min

1. *This page is well organized as it indicates easy categories of learners where you can get the content by grade because it is not in the same way we teach first or second grade*.	5 min
Discussion Question 7	
As I introduced, the goal of this research is to translate the content of Khan Academy into the Kinyarwanda language to increase the accessibility of digital content to a person with disabilities. What are your suggestions on the interface to meet the Rwandan context?	Time
1. *Because most of our children cannot follow the formal education system, it would be better to use the examples and images found in our families.*<br>2. *I find the educational content can be supportive in the learning of children with Autism, but there are some changes to be made to adapt this content to our local context.*	5 min
Discussion Question 8	
What are the challenges you find to be addressed before using this interface?	
1. *As you can see, it is not possible to play YouTube videos in this area, I encourage to do advocacy for us to get high-speed Internet in the rural area as we see in cities of Rwanda; however, if there is a possibility to download the video and play it offline can support our education system.*	
Discussion Question 9	
If the content is translated into Kinyarwanda, what are elements can be included in the interface to help children with Autism to stay focused.	Time
1. *When the content is translated into the local language together with examples found in our environment, it will be helpful to all children, including those considered to have normal intellectual capacity*.	5 min
Discussion Question 10	
Teaching children with Autism in class, we need reward actions for them. How do you reward the children in the class to encourage them in their learning?	Time
1. *As we said, learners with Autism need rewards like giving them a pen or a toy to encourage them to study; here, teachers don't have various tangible rewards to offer. While teaching, the student with ASD can accomplish the given task in a good way.*"<br>2. *In our class, we use an object that is available such as pens, and books as a reward to motivate children with Autism.*	5 min
**Additional Comments and Reflections**	
1. *When the content is translated into the local language together with examples found in our environment, it will be helpful to all children, including those considered to have normal intellectual capacity*.<br>2. *Availability of offline content will also be supportive of teaching children with Autism in and outside the class*.<br>3. *Participation of educators in the design and development of digital content for children with Autism can improve the quality of education for children with Autism*.	5 min
